# Constrained spherical deconvolution analysis of the limbic network in human, with emphasis on a direct cerebello-limbic pathway

**DOI:** 10.3389/fnhum.2014.00987

**Published:** 2014-12-08

**Authors:** Alessandro Arrigo, Enricomaria Mormina, Giuseppe Pio Anastasi, Michele Gaeta, Alessandro Calamuneri, Angelo Quartarone, Simona De Salvo, Daniele Bruschetta, Giuseppina Rizzo, Fabio Trimarchi, Demetrio Milardi

**Affiliations:** ^1^Department of Biomedical Sciences and Morphological and Functional Images, University of MessinaMessina, Italy; ^2^Department of Neurosciences, University of MessinaMessina, Italy; ^3^IRCCS Centro Neurolesi Bonino PulejoMessina, Italy

**Keywords:** CSD, probabilistic tractography, cerebello-limbic pathway, cerebellum, hippocampus, limbic system, quantitative analysis, MRI

## Abstract

The limbic system is part of an intricate network which is involved in several functions like memory and emotion. Traditionally the role of the cerebellum was considered mainly associated to motion control; however several evidences are raising about a role of the cerebellum in learning skills, emotions control, mnemonic and behavioral processes involving also connections with limbic system. In 15 normal subjects we studied limbic connections by probabilistic Constrained Spherical Deconvolution (CSD) tractography. The main result of our work was to prove for the first time in human brain the existence of a direct cerebello-limbic pathway which was previously hypothesized but never demonstrated. We also extended our analysis to the other limbic connections including cingulate fasciculus, inferior longitudinal fasciculus, uncinated fasciculus, anterior thalamic connections and fornix. Although these pathways have been already described in the tractographic literature we provided reconstruction, quantitative analysis and Fractional Anisotropy (FA) right-left symmetry comparison using probabilistic CSD tractography that is known to provide a potential improvement compared to previously used Diffusion Tensor Imaging (DTI) techniques. The demonstration of the existence of cerebello-limbic pathway could constitute an important step in the knowledge of the anatomic substrate of non-motor cerebellar functions. Finally the CSD statistical data about limbic connections in healthy subjects could be potentially useful in the diagnosis of pathological disorders damaging this system.

## Introduction

The human limbic system is an intricate network involved in emotion, motivation and social behavior (Mega et al., 1997). It is important also for the storage of memory associated with emotional events. The limbic system consists of several cortical and subcortical structures, with the latter including amygdala, hippocampus, hypothalamus, mammillary bodies, ventral striatum and some thalamic nuclei including anterior, intralaminar, and medial dorsal groups (Catani et al., 2013). All these structures, as well as their several interconnections including the fornix, mammillo-thalamic tract (bundle of Vicq D’Azyr), anterior thalamic connections, cingulate gyrus and uncinate gyrus (Testut and Latarjet, 1948; Brodal, 1981; Catani et al., 2013), are connected with cortical areas.

The current view provides a division of the limbic system into three different functional-anatomical networks, partially overlapping. The first network, dedicated to memory and spatial orientation, consists of a hippocampal-diencephalic circuit and a parahippocampal-retrosplenial circuit, involving fornix, mammillo-thalamic tract and ventral cingulum (Aggleton, 2008; Vann et al., 2009). The second one, supporting visceral and emotional states integration with cognition and behavior, is the temporo-amygdala-orbitofrontal network, connected by uncinated fasciculus (Mesulam, 2000). The third one is the dorsomedial default-mode network, consisting of several medial regions integrating inherent brain’s functionality (Raichle and Snyder, 2007). Two key structures of the limbic system are the retrosplenial cortex and the anterior thalamic nuclei that are considered important nodes for information integration, memory, visual-spatial processes and proprioception (Vann et al., 2009; Jankowski et al., 2013). These functions are possible due to the co-operative actions between hippocampus, anterior thalamic nuclei, dorsolateral prefrontal cortex, parietal cortex, occipital cortex and, as recently proposed, the claustrum (Maddock, 1999; Vann et al., 2009; Milardi et al., 2013). Moreover, although traditionally the role of the cerebellum was considered mainly associated to motion control, recent studies highlighted a cerebellar involvement in cognitive regulation. Several cerebellar-cerebral pathways are likely to be involved in emotional behavior, learning skills, mnemonic and behavioral processes (Peng et al., 2013). In addition, growing evidence supports the hypothesis of a cerebellar key role in limbic functions, since it is involved in spatial navigation and motor learning. While functional magnetic resonance imaging (fMRI) studies detected several cortico-cerebellar networks in human brain (Habas et al., 2009; O’Reilly et al., 2010; Buckner et al., 2011), demonstrating non-motor cerebellar role, cerebellar connectivity with the limbic system was not specifically investigated. Although these studies suggest the existence of a direct cerebello-limbic connection, quoting Rochefort et al. “a direct cerebello hippocampal projection remains to be discovered” (Rochefort et al., 2013). Therefore, a thorough cerebellar-limbic network map could be an advance in the comprehension of the cerebellar role in emotion elaboration (Baumann and Mattingley, 2012).

On these basis we searched and detected for the first time in human brain a direct cerebello-limbic pathway by means of probabilistic Constrained Spherical Deconvolution (CSD) tractography. At the same time, in order to support the reliability of our results, we further extended our analysis to the other limbic connections providing a CSD-based analysis also of cingulate fasciculus, inferior longitudinal fasciculus, uncinated fasciculus, anterior thalamic connections and fornix. Indeed probabilistic CSD tractography represents a method able to overcome many limitations of other Diffusion Tensor Imaging (DTI) techniques and is able to provide more accurate data (Jeurissen et al., 2011).

## Materials and methods

### Participants

Fifteen healthy subjects with no history of neurological diseases (8 males, 7 females; age range 25–32 years; mean age 29) were recruited. Each subject wrote informed consent and the entire study was approved by Ethical Committee of “I.R.C.C.S.—Istituto di Ricovero e Cura a Carattere Scientifico—Centro Studi Neurolesi Bonino-Pulejo, Messina, Italy”, which confirmed that all examinations are in conformity with relevant regulatory standard.

### Data acquisition and pre-processing of diffusion weighted data

The study was performed with a 3T Achieva Philips scanner (Philips healthcare, Best, Netherlands); using a Quasar Dual gradient system (mode 1: 40 mT/m and 200 mT/m/ms; mode 2: 80 mT/m and 100 mT/m/ms), with a 32-channels SENSE head coil. For anatomical comparison and segmentation we used the following MRI sequences:

1. T1-weighted 3D high-resolution Fast Field Echo (FFE) sequence with: TR 25 ms; TE 4.6 ms; flip angle 30°; FOV 240 × 240 mm^2^; reconstruction matrix 256 × 256; voxel size 1 × 1 × 1 mm; slice thickness 1 mm. The acquisition time was 6 min;
2. T2 weighted 3D high resolution Turbo Spin Echo (TSE) with: TR 2500 ms; TE 380 ms; FOV 250 × 250 mm^2^; reconstruction matrix 312 × 312; voxel size 0.8 × 0.8 × 0.8 mm; slice thickness 0.8 mm. The acquisition time was 9 min and 38 s. The use of 3D TSE sequence permitted to obtain high-resolution images with a relative short acquisition time.

For tractography we used:

3. Diffusion-weighted (DW) MRI with a dual phase encoded pulsed gradient spin echo sequence; 60 gradient diffusion directions were used in order to improve correction of susceptibility and Eddy’s currents distortion (Embleton et al., 2010) following the rules stated by an electrostatic repulsion model (Jones et al., 1999). A SENSE factor of 2 was used.

We used these sequence parameters: b-value of 1000 s/mm^2^; TR 11884 ms; TE 54 ms; FOV 240 × 240 mm^2^; scan matrix 112 × 112; reconstruction matrix 256 × 256; slice thickness 2 mm without inter-slice gap. This sequence was repeated four times for each patient in order to correct subject motion and induced Eddy currents. B-matrix was adjusted in order to obtain a better modulation of DW images (Leemans and Jones, 2009) with the Jacobian of the transformation matrix (Jones and Cercignani, 2010). The total acquisition time was 25 min.

### Tractography

We used probabilistic CSD, that is a modified High Angular Resolution Diffusion Imaging (HARDI) technique. Constrained Spherical Deconvolution estimates, directly from the DW signal, the fiber Orientation Distribution Function (fODF) by means of positive spherical deconvolution (Tournier et al., 2007). We set to 8 the degree of spherical harmonics to achieve robustness to noise. From fODF, obtained by deconvolution of a single fiber DW signal response, it was possible to find the components with specific orientation.

The use of CSD-based method to extract local fiber orientations allows to overcome several limitations of other commonly used tractographic techniques, such as DTI (Tournier et al., 2007; Jones and Cercignani, 2010).

We applied a directionally encoded color map indicating the principal diffusion directions (Pajevic and Pierpaoli, 1999). In particular, red, green and blue colors indicate respectively left–right, anterior–posterior and superior-inferior patterns, varying pureness and intensity according to fibers directions. Probabilistic streamline fiber tracking was performed by calculating fODF peak direction closest to the previous stepping direction (as stated by Newton’s method in optimization) using trilinear interpolation. Tractography was performed following these settings: maximum fiber length 100 mm, maximum angle = 10°, step size = 0.2 mm, minimal fODF amplitude = 0.15 (this choice, even if underestimates fibers tracts, represents a more conservative approach that is able to reduces false positive reconstructions) (Descoteaux et al., 2009; Tournier et al., 2011). It is known higher b-values permit a smaller angles among fibers (Alexander and Barker, 2005; Tournier et al., 2007), but the latter increases difficulty to correct eddy currents and motion artifacts, thus we chose a lower b-value in order to obtain better correction. Furthermore, according to an anatomical model-based approach, we selected regions of avoidance (ROAs) that filter out tracts, in order to improve fiber tracking. The combined use of ROIs and ROAs allowed us to obtain more reliable selection of tracts of interest.

### Segmentation and brain areas identification

All MRI data were spatially normalized to Montreal Neurological Institute (MNI) stereotactic space using the SPM8 segmentation toolbox^1^. Segmentation was manually performed by one expert rater using Analyze 11.0 (AnalyzeDirect, Inc., Kansas City, Kansas, USA) as follows: first, the individual volumes obtained from the T1 and T2 sequences were opened into the viewer; second, the contrast values were set to maximally increased visibility of each brain structure; third, the axial view was magnified to make easier the individuation of the cerebellar mask.

The fastigial nucleus was segmented by using MNI coordinates and following data provided by other authors (Dimitrova et al., 2002; Park et al., 2014).

In addition, anterior thalamic nuclei, amygdala and hippocampus were also segmented. The hippocampal subregions (CA1, Fimbria, Subiculum and Presubiculum) were detected following the study of Frisoni et al. (2008).

### Quantitative analysis

Quantitative analysis was performed with Explore DTI (Leemans et al., 2009); we considered tracts number, tracts volume, tracts length mean, Fractional Anisotropy (FA) and Apparent Diffusion Coefficent (ADC). The study of the FA right-left variability was possible after the CSD data transfer to diffusion spectrum imaging (DSI) Studio^2^, following the instruction provided by the documentation.

### Intra- and inter-subjects variability

In order to assess intra- and inter-subjects variability of tracts number (number of streamlines = N.), we calculated a lateralization index (Parker et al., 2005; Lebel and Beaulieu, 2009) according to the following formula: (N. Right − N. Left)/(N. Right + N. Left). In addition we also assessed FA right-left variability. Statistical significance of the inter-subjects and intra-subjects variability was determined using a 2-tailed *t*-test; *P* values <0.05 were considered statistically significant.

## Results

**Figure 1:**
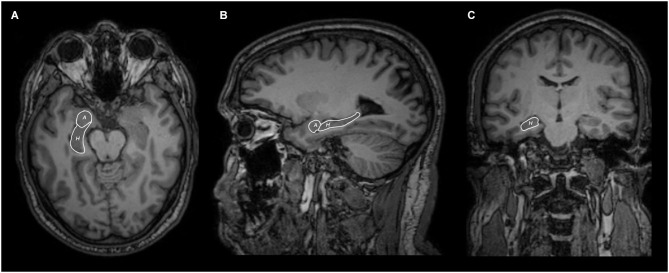
**Axial (A), sagittal (B) and coronal (C) MRI views of hippocampus (H) and amygdala (A) segmentation**.

**Figure 2:**
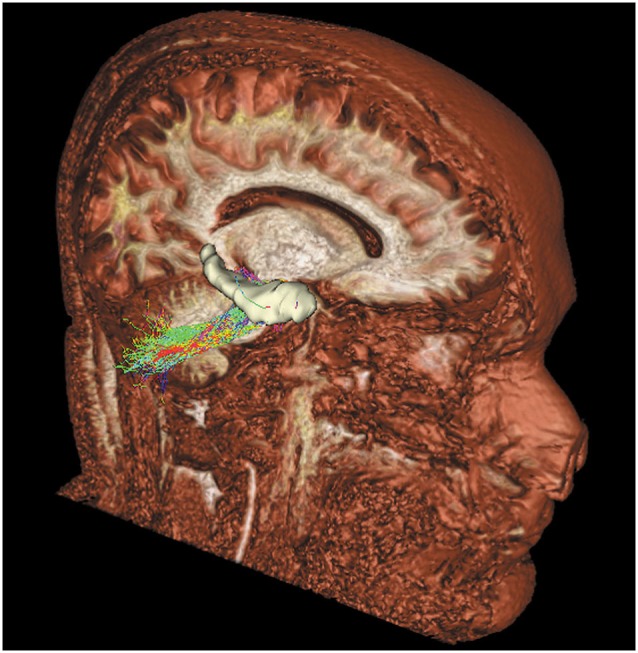
**Tridimensional sagittal view of a right cerebello-limbic direct pathway**.

In 15 out of 15 subjects we segmented the hippocampus (Figure 1) and bilaterally traced fiber bundles passing through the superior cerebellar peduncles, linking the hippocampus and the cerebellum: a direct cerebello-limbic pathway (Figure 2). Mean quantitative analysis of this pathway is shown in Table 1. No statistically significant differences were found comparing intra- and inter-subjects right and left variability for tracts number, tracts volume, tracts length mean, FA and ADC (*p* > 0.05).

**Table 1:** **Mean quantitative analysis of the direct cerebello-limbic pathway for each subject**.

Subject number	Number of tracts	Tract length mean (mm)	Tracts volume (mm^3^)	FA	ADC (× 10^−3^ mm^2^/s)
1	32	179.257	3149.5	0.421575	0.863082
2	31	152.657	3036.13	0.434396	0.853043
3	34	292.244	3089.38	0.464008	0.891949
4	30	179.209	2935.63	0.427374	0.842468
5	29	132.097	3025.76	0.435526	0.980773
6	33	139.587	3925.36	0.403828	0.924584
7	32	250.141	3412.85	0.468012	0.911252
8	31	221.912	3004.21	0.421548	0.861254
9	32	130.922	2998.14	0.412985	0.854698
10	33	198.775	3662.52	0.409982	0.932225
11	35	189.119	2988.77	0.432815	0.872011
12	38	129.176	3088.87	0.454113	0.960423
13	30	140.187	3115.13	0.467812	0.862458
14	29	260.243	3142.9	0.428127	0.862145
15	34	225.872	3181.24	0.433466	0.912293
Mean ± SD	32.2 ± 2.4	188.09 ± 52.4	3183.75 ± 276.4	0.434 ± 0.02	0.892 ± 0.04

**Figure 3:**
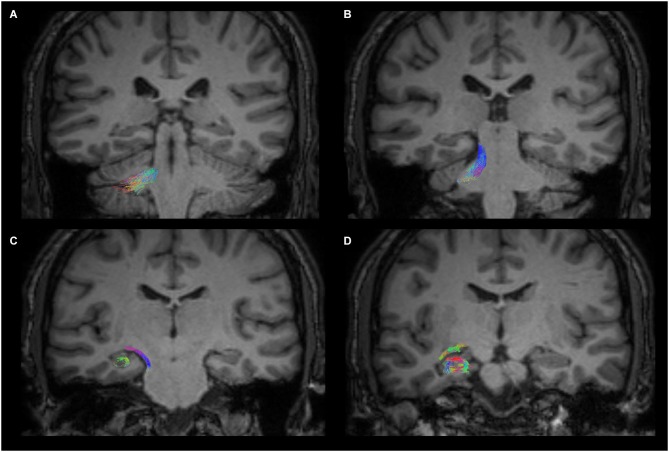
**Anatomical course of the cerebello-limbic direct pathway.** The coronal images show the course of a right cerebello-limbic pathway, starting from the cerebellum **(A)**, proceeding through the superior cerebellar peduncle **(B)**, reaching the temporal lobe **(C)** and the hippocampus **(D)**.

Figure 3 shows the anatomical course of the cerebello-limbic pathway. It spreads from the cerebellar cortex to the medial part of the cerebellum (Figure 3A) and it passes through the superior cerebellar peduncle (Figure 3B). Then the bundle goes medial and laterally underneath the thalamus and the lenticular nucleus (Figure 3C), reaching the medial temporal lobe through the white matter at the margin of the inferior horn of the lateral ventricle. Tracked streamlines pass cranially to the temporal horn, move interspersed to the medial fibers of the inferior longitudinal fasciculus and finally reach the hippocampus at several levels (Figure 3D).

**Figure 4:**
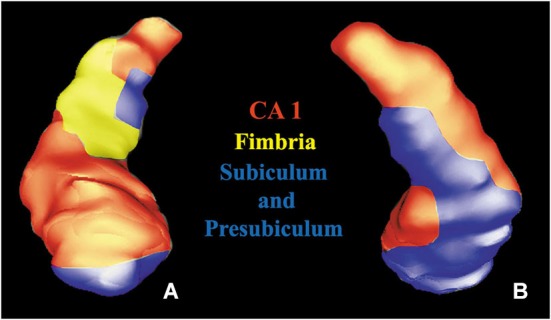
**Volume rendering of a hippocampus**. Anterior **(A)** and posterior **(B)** views of a segmented hippocampus with manually colored subregions: CA1 (red), subiculum and presubiculum (blue) and fimbria (yellow).

**Figure 5:**
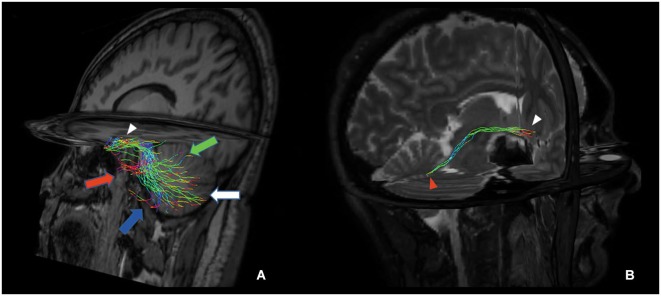
**Tridimensional multiplanar view of a direct cerebello-limbic pathway**. An oblique sagittal view depicts that the large part of the fibers connects the vermis (green arrow), Crus I (red arrow), Crus II (blue arrow), lobules VIII, IX and X (white arrow) with the hippocampus (white arrowhead) **(A)**. In a right targeted parasagittal view the isolated connection between hippocampus (white arrowhead) and fastigial nucleus (red arrowhead) can be seen **(B)**.

The hippocampus was widely interested by this white matter fiber pathway. After the detection of hippocampal subregions (Figure 4), we found the involvement of CA1, fimbria, subiculum and presubiculum. In the cerebellum we found that the fibers predominantly reached the vermis, lobules VIII, IX, X, Crus I, Crus II (Figure 5A) and fastigial nucleus (Figure 5B).

We extended our analysis to the amygdala, separately considered as streamline seed, setting hippocampal segmentation as ROA. We did not find streamlines between amygdala and the cerebellum.

**Figure 6:**
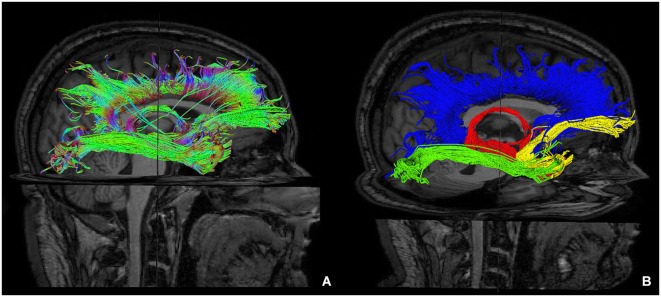
**Reconstruction of main limbic pathways, with involvement of prefrontal, frontal, parietal, occipital and temporal cortices (A)**. Each pathway was colored manually after morphological isolation, in order to show them separately: cingulate fasciculus (blue), uncinate fasciculus (yellow), fornix (red) and inferior longitudinal fasciculus (green) **(B)**.

**Figure 7:**
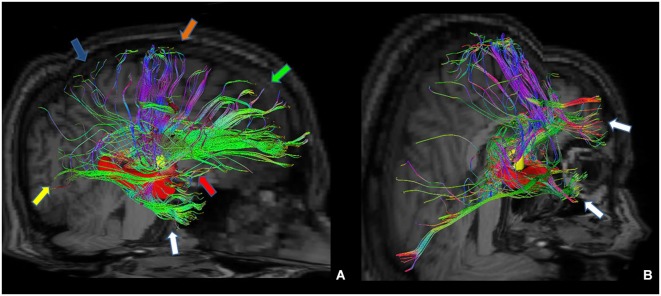
**Tractographic reconstruction of right anterior thalamic pathway after the placement of a ROI corresponding to right anterior thalamic nuclei (yellow ROI)**. This pathway **(A)** is composed by seven white matter bundles which are connected respectively to: cingulate gyrus and prefrontal cortices (green arrow), frontal and secondary motor cortices (orange arrow), parietal cortex (blue arrow), occipital cortex (yellow arrow), temporal cortex (white arrow), mammillary bodies (red arrow) and hippocampus (red ROI). The posterior view **(B)** shows better temporal projections (white arrows).

Furthermore, we isolated the following white matter limbic pathways: cingulate fasciculus, uncinate fasciculus, inferior longitudinal fasciculus, fornix (Figure 6) and anterior thalamic pathway, which is made by four main fiber bundles orientated toward: prefrontal cortex, cingulate gyrus, mammillary bodies, parietal cortex, temporal cortex, occipital cortex and hippocampus (Figure 7).

We assessed intra- and inter-subjects right-left variability also for these pathways looking at the following data: tracts number, tracts length mean, tracts volume, FA and ADC values. Table 2 shows the mean quantitative analysis of all volunteers. No differences were found between subjects (inter-subjects variability *p* > 0.05) for all parameters.

**Table 2:** **Mean quantitative analysis for all subjects of main pathways of the limbic network**.

Pathway	Number of tracts	Tract length mean (mm)	Tracts volume (mm^3^)	FA	ADC (× 10^−3^ mm^2^/s)
cingulate fasciculus right	271	114.203	13375.5	0.481955	0.836845
cingulate fasciculus left	263	115.114	12846.4	0.482246	0.829684
fornix right	181	149.255	9898	0.418114	0.983218
fornix left	179	147.956	9412	0.414618	0.978468
inferior longitudinal fasciculus right	309	122.887	11937.6	0.496579	0.852989
inferior longitudinal fasciculus left	304	123.634	10897.6	0.489165	0.871656
uncinate fasciculus right	326	110.634	8673	0.471165	0.831825
uncinate fasciculus left	265	112.163	7249	0.469612	0.839562
anterior thalamic pathway right	512	104.791	14050.8	0.439577	1.01874
anterior thalamic pathway left	492	98.9746	13352.5	0.410987	1.03377

**Figure 8:**
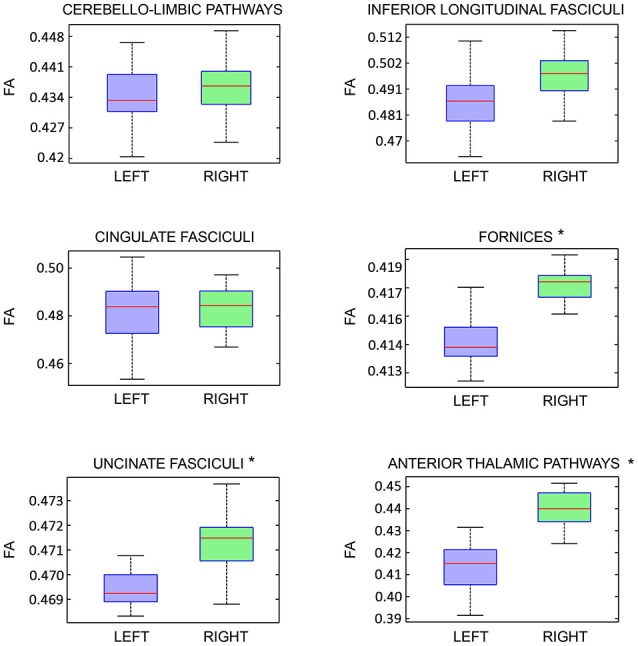
**Fractional Anisotropy (FA) right-left intra-subject variability analysis of each limbic pathway, showed as boxplots**. Statistical significance is indicated by asterisks (*p* < 0.05).

On the other hand, analyzing the FA of each previously described pathway, we detected a significant intra-subjects variability only for the following tracts: fornices, uncinate fasciculi and anterior thalamic pathways with right predominance (Figure 8).

## Discussion

Cerebellum was traditionally associated with motion control, however neural substrate for a cerebellar influence over non-motor functions is becoming even more asserting. Other authors advanced the hypothesis of an internal cerebellar topography where regions of the vermis could be considered the “limbic cerebellum” involved in the modulation of emotions and social behaviors, based on vermis connections with the limbic brain structures (Schmahmann, 1991, 1996, 2000; Stoodley and Schmahmman, 2009). Particularly, cognitive and limbic regions of cerebellum were located in the posterior lobe (lobule VI, VIIA, VIIB including Crus I and Crus II, and possibly lobule IX), with cognitive areas situated laterally whereas autonomic/affective/limbic functions were represented in the vermis. fMRI studies provided evidences in healthy subjects of the activation of different cerebellar sub-regions during fMRI tasks of anger, disgust, happiness, sadness and fear, detecting partial superimposition of these activations, respectively during tasks of anger and fear (Crus I and paravermal lobules VI), disgust and anger (lobule IX), sadness and happiness (lobule VIIIA) (Baumann and Mattingley, 2012). Indeed, transcranial cerebellar direct current stimulation (tcDCS), performed in humans, was able to modulate several cerebellar activities concerning learning, motor control and emotional states integration. Moreover, tcDCS influenced the interactions between the cerebellum and other brain structures induced by transcranial magnetic stimulation (TMS), concerning the control of several functions such as working memory, walking adaptation, and emotional processes (Ferrucci and Priori, 2013). Cerebellum and hippocampus interact also during spatio-temporal prediction of movements, acquisition and long-term storage of motor associations (Onuk et al., 2013; Thieme et al., 2013). Furthermore, it was found that patients with cerebellar cognitive affective syndrome (CCAS) can suffer from emotional altered status, not motivated crying or laughing, and affective changes (Levisohn et al., 2000; Rapoport et al., 2000; Steinlin et al., 2003; Parvizi and Schiffer, 2007; Parvizi et al., 2007), suggesting that the existence of cerebello-limbic connections may allow the emotional processing modulation.

Some evidences exist about a direct anatomical link between the hippocampus and the cerebellum. In cats, rats and monkeys, direct connections between fastigial nucleus and hippocampus were demonstrated (Heath and Harper, 1974; Snider and Maiti, 1976; Heath et al., 1978; Newman and Reza, 1979). Recently a study combining retrograde tracing and degeneration analysis after hippocampal lesion demonstrated a direct projection from the hippocampus to the cerebellum (folia VI–VIII) in chicken (Liu et al., 2012). Our results are in keeping with Liu’s data. In addition, we were able to show the connection between hippocampus and fastigial nucleus that was previously seen in monkey (Figure 5B; Heath and Harper, 1974).

The existence of a direct cerebello-limbic pathway might overcome the objection of Strick et al. (2009) who argued that a big criticism about the hypothesis of a cerebello-limbic functional interaction was the lack of a defined anatomical substrate allowing the connection between the cerebellar output and the limbic system; indeed to date there are no sufficient anatomical literature findings supporting that all the effects on behavior be induced by cerebellar stimulation (Strick et al., 2009). Baumann and Mattingley argued that cerebellar connectivity with limbic networks wasn’t deeply investigated, although fMRI studies demonstrated several cortico-cerebellar connections in humans (Habas et al., 2009; O’Reilly et al., 2010; Buckner et al., 2011; Baumann and Mattingley, 2012) confirming its role in non-motor processes. Finally Rochefort et al. (2013) suggested that many data support the hypothesis that a direct connection between cerebellum and hippocampus exists, despite the fact that it was never demonstrated.

In our study we detected and analyzed for the first time in human brain a direct cerebello-limbic pathway with probabilistic CSD. This pathway consists of a white matter bundle connecting the hippocampus and the cerebellum passing through the superior cerebellar peduncle. Main cerebellar areas reached by this pathway are vermis, lobules VIII, IX, X, Crus I, Crus II and fastigial nucleus. Quantitative comparison showed that there are no statistically significant differences in shape and size of this white matter bundle between each subject analyzed. The comparative analysis of right and left bundles showed no significant differences, with good right-left symmetry for each subject.

Extending our analysis to the amygdala, we did not find direct connections between this structure and the cerebellum. This might be due to the different functional skills of the amygdala, which has a central role in emotions and social behavior control (Phelps and LeDoux, 2005). Moreover amygdala is involved in higher order functions, such as working memory and attention, through defined bidirectional connections with several sovratentorial brain areas (Schaefer and Gray, 2007). Although emotions may induce both sensory and motor effects, whom may also be due to the close relationship between amygdala and motor areas (Cardinal et al., 2002), it might be possible that specific motor skills of the amygdala do not necessarily require a direct connection with the cerebellum.

For the present study we studied main pathways of the limbic system (cingulate fasciculus, inferior longitudinal fasciculus, uncinated fasciculus, anterior thalamic connections and fornix) using probabilistic CSD technique, in order to overcome intrinsic limitation of DTI and to obtain more accurate data. Indeed, recently Kristo et al. (2013) showed that CSD tractography is more reliable than traditional DTI in the white matter tracts reconstruction and analysis.

Although other studies described the white matter connections of the limbic system (Mega et al., 1997; Catani et al., 2013), to our knowledge no previous reconstruction and statistical analysis of these fiber bundles using probabilistic CSD in human were reported in the literature.

It is known that CSD-based technique overcomes several DTI limitations, such as partial volume effects (Jones and Cercignani, 2010), and improves tractographic reconstruction in comparison with common DTI approaches. Other techniques, such as DSI and Q-ball imaging (QBI) are able to obtain similar results compared to CSD; nevertheless, for a clinical use, DSI is less adopted due to its longer scan time (Tournier et al., 2007). On the other hand, although QBI be a powerful technique (Gigandet et al., 2013) with comparable scan time respect to CSD, it suffers from reconstruction limitation of crossing fibers for crossing angle smaller than 45° (Tournier et al., 2008). Therefore, to date, probabilistic CSD is probably the most reliable practical option for clinical use.

Concha et al. (2005) described the cingulate fasciculus and fornix calculating streamlines with deterministic DTI tractography using a 1.5 T MRI scanner. Similarly, Santillo et al. (2013) in a more recent study used deterministic DTI tractography on a 3T MRI scanner to evaluate cingulate fasciculus. A more extensive and complete deterministic DTI reconstruction and statistical analysis of main limbic pathways was published by Pugliese et al. (2009). However it is well known that this approach is unsuitable to reveal kissing, crossing and bridging fibers, thus causing potential underestimation of the data and other pitfalls (Tournier et al., 2007). Recently probabilistic fiber tracking was used to analyze the fornix (Jang and Kwon, 2013, 2014); however these studies were conducted with a 1.5 T MRI scanner and using a not CSD-based probabilistic algorithm. Only Emsell et al. (2013) used CSD tractography to analyze cingulate fasciculus and fornix in human brain, but with a deterministic approach.

Our study provided a detailed analysis of cingulate fasciculus, inferior longitudinal fasciculus, uncinated fasciculus and fornix. In addition we obtained statistical data about anterior thalamic pathways that are known to be extensively connected with hippocampus and several other brain structures as cingulate gyrus, prefrontal cortex, occipital cortex, temporal cortex, parietal cortex and mammillary bodies (Aggleton and Brown, 1999; Vann and Aggleton, 2004; Jankowski et al., 2013). These observations about anterior thalamic pathways support the hypothesis that these nuclei are a critical node in an “extended hippocampal system”. Other findings demonstrated that an anterior thalamic lesion stops synaptic plasticity and takes a role in the etiology of the posterior cingulate hypoactivity, for example in Alzheimer’s Disease (Garden et al., 2009). Moreover anterior thalamic lesions cause altered responses in different tasks concerning spatial memory (Aggleton et al., 1996; Mair et al., 2003).

With respect to right-left comparison, some authors described a number of tracts asymmetry of the uncinate fasciculi, anterior thalamic pathways and fornices (Supprian and Hofmann, 1997; Axer et al., 1999; Highley et al., 2002). Interestingly, although we did not find statistically significant lateralization index for these tracts, we found a significant right-left FA variability for the same tracts (Figure 8). This apparent paradox might be explained by the evidence that FA asymmetries are not influenced by the numbers of streamlines, reflecting mainly microscopic white matter differences (Takao et al., 2013). There is not an unique explanation regarding right-left FA asymmetry. It was advanced the hypothesis that right-left FA differences might be related to interhemispheric differences regarding brain functions, axons diameter and their numerosity, as well as fiber mielination (Thiebaut de Schotten et al., 2011; Takao et al., 2013).

Unfortunately, according to Takao et al. (2013) data about FA asymmetry “are somewhat inconsistent”, therefore further studies involving larger number of subjects comparing different technical approaches should be carried out to clarify the issue.

In conclusion, our study addressed two main goals: first, the demonstration of a direct cerebello-limbic pathway in human brain, and second, the morphological and statistical analysis by probabilistic CSD of major white matter pathways involved into limbic network. Both these findings might be potentially useful to explore pathological conditions damaging this system. However, further studies need to be performed in order to clarify the physiological role of this new direct connection and its involvement in pathological conditions.

### Limitations

It is known that tractography suffers from inherent technical limitations. For example, DTI tractography is usually less reliable to accurately analyze reconstructed tracts (Jones and Cercignani, 2010). We reduced this limitation by using probabilistic CSD. In addition, the directionality (afferent-efferent) of the connections cannot be evaluated (Chung et al., 2011; Parker et al., 2013). Finally, reconstruction may be error-prone both during acquisition and postprocessing since multiple artifacts and false positive tracts might be produced by inaccurate reconstruction (Jones and Cercignani, 2010); however the use of restrictive technical choices with respect to usual standards (see Section Materials and Methods), allowed us to reduce this limitation at the cost of potential underestimation of fibers bundles (Descoteaux et al., 2009; Tournier et al., 2011).

## Author’s contributions

Alessandro Arrigo: study concepts/study design, data acquisition, data analysis, data interpretation; Enricomaria Mormina: study concepts/study design, data acquisition, data analysis, data interpretation; Giuseppe Pio Anastasi: Guarantor of integrity of entire study, approval of final version of submitted manuscript; Michele Gaeta: study concepts/study design, data acquisition, data analysis, data interpretation; Alessandro Calamuneri: statistical analysis, manuscript revision; Angelo Quartarone: Guarantor of integrity of entire study, approval of final version of submitted manuscript; Simona De Salvo: data acquisition; Daniele Bruschetta: literature research, manuscript revision; Giuseppina Rizzo: literature research, manuscript revision; Fabio Trimarchi: literature research, manuscript revision; Demetrio Milardi: study concepts/study design, manuscript revision.

## Conflict of interest statement

The authors declare that the research was conducted in the absence of any commercial or financial relationships that could be construed as a potential conflict of interest.

## Footnotes

^1^www.fil.ion.ucl.ac.uk/spm

^2^http://dsi-studio.labsolver.org
